# Urokinase Plasminogen Activator Receptor (uPAR) and Plasminogen Activator Inhibitor-1 (PAI-1) Are Potential Predictive Biomarkers in Early Stage Oral Squamous Cell Carcinomas (OSCC)

**DOI:** 10.1371/journal.pone.0101895

**Published:** 2014-07-07

**Authors:** Synnøve Magnussen, Oddveig G. Rikardsen, Elin Hadler-Olsen, Lars Uhlin-Hansen, Sonja E. Steigen, Gunbjørg Svineng

**Affiliations:** 1 Department of Medical Biology, Faculty of Health Sciences, University of Tromsø - The Arctic University of Norway, Tromsø, Norway; 2 Department of Otorhinolaryngology, University Hospital of North Norway, Tromsø, Norway; 3 Diagnostic Clinic – Clinical Pathology, University Hospital of North Norway, Tromsø, Norway; Innsbruck Medical University, Austria

## Abstract

Oral squamous cell carcinoma (OSCC) is often associated with metastatic disease and a poor 5 year survival rate. Patients diagnosed with small tumours generally have a more favourable outcome, but some of these small tumours are aggressive and lead to early death. To avoid harmful overtreatment of patients with favourable prognosis, there is a need for predictive biomarkers that can be used for treatment stratification. In this study we assessed the possibility to use components of the plasminogen activator (PA) system as prognostic markers for OSCC outcome and compared this to the commonly used biomarker Ki-67. A tissue-micro-array (TMA) based immunohistochemical analysis of primary tumour tissue obtained from a North Norwegian cohort of 115 patients diagnosed with OSCC was conducted. The expression of the biomarkers was compared with clinicopathological variables and disease specific death. The statistical analyses revealed that low expression of uPAR (p = 0.031) and PAI-1 (p = 0.021) in the tumour cells was significantly associated with low disease specific death in patients with small tumours and no lymph node metastasis (T1N0). The commonly used biomarker, Ki-67, was not associated with disease specific death in any of the groups of patients analysed. The conclusion is that uPAR and PAI-1 are potential predictive biomarkers in early stage tumours and that this warrants further studies on a larger cohort of patients.

## Introduction

Squamous cell carcinoma (SCC) is the most frequent malignant tumour in the oral cavity, with a propensity to early and extensive lymph node metastasis [1]. In most populations, the two major risk factors are tobacco use and alcohol consumption, which seem to function synergistically [2]. Prognosis is mainly determined by the stage of the tumour at presentation [2], which is determined according to the TNM-staging system: tumour size (T), regional lymph node metastasis (N) and distant metastasis (M) [3]. Small tumours without metastasis largely present with good prognosis [4], however, there are substantial individual differences in the response to treatment of patients belonging to the same TNM-groups [3], mainly because of large heterogeneity of the tumours [5]. The relationship between N status and prognosis has been reported by numerous studies and most patients that present with a lymph node metastasis will undergo therapeutic neck dissection [2], [6]. The controversy is whether patients with no lymph node metastasis at diagnosis should be given the same treatment due to high recurrence rate and the large number of occult metastases [7], [8]. Instead, a “watchful waiting” strategy is commonly used to avoid overtreatment of patients. Hence, there is a great need for a better prognostic tool to distinguish between patients with no lymph node metastasis at the time of diagnosis (N0) that are in need for adjuvant treatment, and those that can safely be monitored with a “watchful waiting” strategy.

A long list of prognostic biomarkers has been suggested for OSCC, but there is still a need for identification of new and robust prognostic markers [5], [9]. Some of the most promising were epidermal growth factor receptor (EGFR), p53 and matrix metalloproteinases (MMPs), though conflicting results exist [4], [5], [10]. Also, constituents of the plasminogen activator (PA) system have been suggested as promising biomarkers in OSCC [11], and several proteins of the PA system have been shown to correlate to poor prognosis [12]–[18].

Cancer cells are thought to exploit the PA system and MMPs during cancer invasion, enabling ECM degradation and cell migration [19]. The key effector of the PA system, the serine protease plasmin, is readily activated from its precursor plasminogen, by either urokinase plasminogen activator (uPA) or tissue type plasminogen activator (tPA). tPA is primarily thought to be involved in fibrinolysis, while uPA is mainly involved in wound healing and cancer invasion. The proteolytic activity of uPA is greatly enhanced by binding to its cell surface localized receptor (uPAR) [20], which is often concentrated at the leading edge of migrating cells [21]. Plasminogen activator inhibitor-1 (PAI-1) and PAI-2 are involved in the regulation of uPA and tPA activity [22]. In addition to regulation of proteolysis, both uPAR and PAI-1 have roles directly linked to cell adhesion and migration through their interactions with the extracellular matrix constituent vitronectin [23], [24].

In this study we assessed the possibility to use components of the PA system as prognostic biomarkers for OSCC outcome and compared this to Ki-67 which is a commonly used biomarker in several cancers. The expression of the biomarkers in small tumours with no lymph node metastasis at the time of diagnosis are of particular interest, as this could help distinguish between patients in need of additional treatment and those where less is better.

## Materials and Methods

### Ethics statement

The study was approved by the Regional Committees for Medical and Health Research Ethics, Northern Norway (No. 22/2007). The patient information was anonymized and de-identified prior to analysis. The ethics committee deemed it unnecessary to obtain written or oral consent from the participating patients.

### Patients and specimens

From the archives of the Diagnostic Clinic, University Hospital of North Norway, 160 patients with histologically verified diagnoses of primary SCC of the oral cavity and the oropharynx in the period 1986–2002 were selected. From this group, patients with SCC of the oropharynx and with verrucous tumours, as well as those who had received prior radiotherapy to the head and neck area, were excluded from the study. The remaining specimens represented biopsies and surgical resections (in some cases both) from mobile tongue, floor of the mouth, bucca, gingiva and soft palate from a total of 115 patients. Clinical data and tumour stage according to TNM-classification [25] was retrieved from patients files, pathology reports, Statistics Norway and the Cause of Death Registry. The N and M statuses were determined by clinical and radiological examination. The last day of follow up was January 1^st^, 2012. The normal tissues used as controls were anonymized and obtained from the archives of the Diagnostic Clinic, University Hospital of North Norway.

### Tissue microarray (TMA)

Cores of 0.6 mm were taken from the representative tumour tissue and inserted into a recipient paraffin block to create a tissue microarray, using a Beecher Instruments Micro Tissue Arrayer. Eight cores were taken per tumour and distributed pairwise into a total of four parallel recipient microarray blocks (A, B, C and D). Sections of 4 µm were cut and transferred to Superfrost+ slides for immunohistochemical (IHC) analysis.

### Immunohistochemistry

Sections of the TMA blocks were immunohistochemically stained for the presence of uPAR, uPA, PAI-1, and Ki67. In addition, normal buccal mucosa tissue (n = 5) was stained for uPAR and PAI-1. The antibodies and staining conditions used are listed in table 1. All TMAs were also stained for cytokeratin to verify the presence of epithelial cancer tissue, and cores without such tissue were withdrawn from the evaluation. After deparaffinization in xylene, sections were rehydrated in graded alcohol baths. The staining was performed essentially as previously described [26], with some modifications as described below for the various antibodies. Heat-induced epitope retrieval (HIER) was performed on all sections prior to blocking of endogenous peroxidase activity. All HIER was performed at 95–99°C for 20 minutes in 10 mM citrate buffer pH 6.0. Optimization of pretreatment conditions was performed for each staining. A negative control where the primary antibody was omitted was included for all antibodies used and showed no staining in all cases.

**Table 1:** Primary antibodies used for IHC.

Primary antibodies	Dilution	Wash buffer	Detection
Mouse monoclonal anti-human uPAR (#3936, Sekisui Diagnostica, Stamford, CT, USA)	1∶10, 4°C ON*	Wash buffer A (PBS ^w^/0.41 M NaCl, 0.3% Tween-20, pH 6.0). Assay buffer (PBS ^w^/1% BSA, 0.3% Tween-20, pH 6.0). Wash buffer B (PBS ^w^/0.41 M NaCl, 1% BSA, 0.3% Tween-20, pH 6.0).	EnVision+ Dual Link system HRP (+DAB), for rabbit and mouse primary antibody detection (Dako; Glostrup, Danmark).
Rabbit polyclonal anti-human uPA (Ab24121, Abcam Inc., Cambridge, MA, USA)	1∶75, 4°C ON	PBS.	EnVision+ system HRP (+DAB), for rabbit primary antibody detection (Dako North America, Carpintera, CA, USA).
Rabbit polyclonal anti-human PAI-1 (BT-BS3503, Nordic BioSite, Täby, Sweden)	1∶100, 4°C ON	PBS.	EnVision+ system HRP (+DAB), for rabbit primary antibody detection (Dako North America, Carpintera, CA, USA).
Mouse monoclonal anti-human PAI-1 (#3785, Sekisui Diagnostica Stamford, CT, USA)	1∶10	Wash buffer A (as described above). Assay buffer (PBS^w^/1% BSA, 0.3% Tween-20, pH 7.2). Wash buffer B (as described above).	EnVision+ Dual Link system HRP (+DAB), for rabbit and mouse primary antibody detection (Dako North America, Carpintera, CA, USA).
Anti-Ki67 (790–4286, Ventana Medical systems, Inc., Tucson, AZ, USA)	According to instruction from Ventana.	According to instruction from Ventana.	Ventana iView DAB detection kit (cat.no. 760-09, Ventana Medical systems, Inc., Tucson, AZ, USA).
Anti-Pan-Cytokeratin, AE1/AE2/PCK26 (760–2135, Ventana Medical systems, Inc., Tucson, AZ, USA)	According to instruction from Ventana.	According to instruction from Ventana.	Ventana iView DAB detection kit (cat.no. 760-09, Ventana Medical systems, Inc., Tucson, AZ, USA).

*abbreviations used: ON, overnight; RT, room temperature; HRP, Horseradish peroxidase; DAB, diaminobenzidine; PBS, Phosphate buffered saline; BSA, bovine serum albumin.

### uPAR

Subsequent to peroxidase blocking, sections were washed in wash buffer A (Table 1). Unspecific antigen binding was blocked using assay buffer (Table 1) with 10% goat serum (Dako North America, Carpintera, CA, USA), which was also used for primary antibody dilution. Sections were then washed in wash buffer A, before the primary antibody was added. Subsequent washing was performed in wash buffer B (Table 1) before the primary antibody was detected (Table 1). The specificity of the monoclonal anti-human uPAR antibody #3936 has been validated in several studies [27]–[29], including using preadsorption of the antibody with recombinant native soluble form of uPAR [27], or with purified soluble uPAR from phospholipase C treated U937 cells [29], which both resulted in strong reduction in staining of tumour tissue. Carriero et al. also compared the performance of the #3936 antibody to the polyclonal #399 anti-uPAR antibody, and found good agreement between the staining obtained with the two antibodies [28]. In addition, the specificity was validated using our IHC protocol and on Western blotting as described in information S1 and figure S1.

### uPA and PAI-1

PBS ^w^/1.5% goat serum was used for both antibody dilution as well as blocking unspecific antigen binding. The specificity of the anti-uPA antibody (Ab24121) was verified by staining pancreatic cancer (Figure S2) and placenta tissue (data not shown) which are known to be positive for uPA [30]–[32], and the specificity of the anti-PAI-1 antibody (BT-BS3503) used was verified by staining human placenta tissue (Figure S3) [32], [33]. Descriptions of the methods used and results are presented in information S1.

### Ki-67 and Cytokeratin

The staining for both Ki-67 and Cytokeratin were performed at the Diagnostic Clinic-Clinical Pathology at the University Hospital of North Norway using the Ventana BenchMark XT automated slide preparation system (Ventana Medical Systems, Inc., Tucson, AZ). The accredited procedures were performed according to the ISO/IEC 15189 standard.

### Scoring methods

Several parallel cores from each tumour were stained. However, for some markers, not all cores could be scored due to technical issues, or due to the lack of tumour tissue, and were therefore not included in the analysis. The mean number of evaluated cores per marker was 4.75 for uPAR, 3.01 for uPA, 3.51 for PAI-1 and 2.00 for Ki-67. The scoring of the uPAR, uPA and PAI-1 staining was semi-quantitative [34], [35], and only cytoplasmic and cell membrane staining was recorded. The staining index (SI) was calculated as a product of staining intensity (none (0), weak (1), moderate (2) or strong (3)), and proportion of positive tumour cells (none (0), <10% (1), 10–50% (2), 51–80% (3) or >80% (4)). Thus, the SI for each core differed from a minimum value of zero to a maximum of 12. Each patient's final score for each marker was the mean SI of all cores evaluated. Scoring of the uPAR and PAI-1 staining of the normal buccal mucosa tissue was performed in the same manner as for the tumour tissue. Ki-67 was scored in a modified version as percentage of nuclei stained; 1 (<10%), 2 (10–50%), 3 (>50%) [36]. All slides were scored by one pathologist (SES) and one head and neck surgeon (OR). There was a good agreement between observers as Spearman's Rho correlation coefficient was 0.753 and 0.881 (p<0.001) when tested on uPA and uPAR scorings in random samples in 25% of the cases. A correlation of deviation between the cores was 33.8% for uPA which reflects the heterogeneity of the tumours.

### Statistical analysis

The analyses were performed by using IBM SPSS statistics 19 for Windows (IBM Corporation Armonk, NY, USA). Cut-off points were determined to obtain binary variables for statistical analyses and were based on the median value of the final scores for each marker. Values below the median point were designated low-expression, while the values in the upper median part were designated as high-expression. The cut-off value of the evaluated markers was 5.63 for uPAR, 7.30 for uPA, 5.25 for PAI-1, and 2.00 for Ki-67. Associations between different categorical variables were assessed with Pearson's Chi-Square test, and one-way analysis of variance (ANOVA) was used to compare means. Univariate analyses of time from diagnosis to death were performed using the Kaplan-Meier method, and differences between categories were estimated by the log-rank test, with the date of diagnosis as starting point. The multivariate analysis was carried out using the Cox proportional hazards model. The correlation analyses were done using Spearman's Rho (2-tailed) and presented as a Scatter plot with regression line and 95% confidence interval lines. All results were considered significant if p≤0.05, and reported according to the REMARK guidelines by McShane et al. [37].

## Results

### Clinical characteristics in relation to disease specific death

Primary tumour tissue from 115 patients in a North Norwegian cohort diagnosed with OSCC from 1986 to 2002 was analysed in this retrospective study and the clinicopathological variables are listed in table S1. A total of 64 males and 51 females with a median age of 65 years were included in the study. From the official records at Statistics Norway and the Cause of Death Registry it was found that 42% of the male patients and 37% of the females died a disease specific death within 5 years from diagnosis. Mean overall survival was 53.9 months for men and 82.9 for women, and the difference was statistically significant (p = 0.033). The mean disease specific survival was also significantly shorter for men (80.8 months) than for women (128.2 months)(p = 0.019). Most patients presented with moderately or well differentiated tumours which were relatively small (T1 (34%) or T2 (37%)). In addition, 63% (72 out of 115) did not have any detectable lymph node metastasis, indicating that the majority were diagnosed at an early disease stage.

As expected, we found that tumour size (T-status) and lymph node status correlated with both overall (data not shown) and disease specific death (Table S1). In early stage disease, T1 vs T2–T4 and N0 vs N+/unknown, showed that both of these variables were significant in the multivariate analyses, with Hazard Ratio 2.665 (95% CI 1.224–5.804) for T-stage (p = 0.007), and 2.633 (95% CI 1.425–4.865) for N-stage (p = 0.002).

### Staining patterns

**Figure 1:**
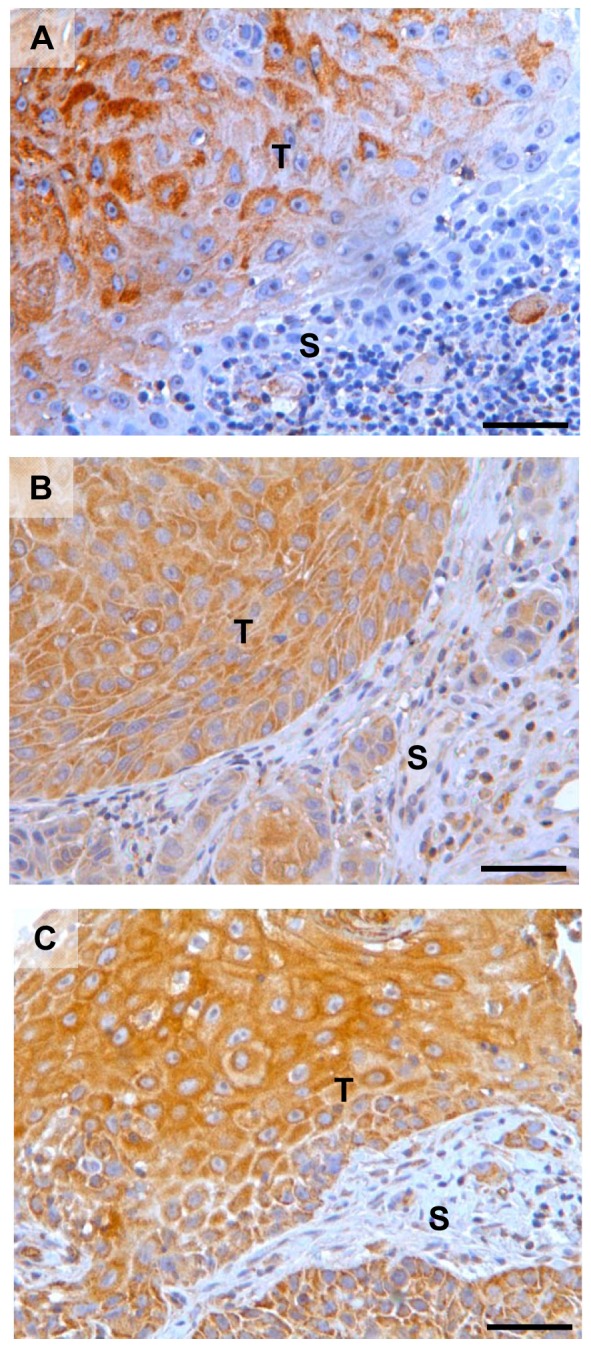
Staining pattern for uPAR, PAI-1 and uPA in OSCC. Representative photomicrographs of tissue microarray sections stained for the markers **A**) uPAR, **B**) PAI-1 and **C**) uPA. Positive staining is seen as brown colour, nuclei are stained blue with haematoxylin. Scalebar = 50 µm. T = Tumour. S = Stroma.

Immunohistochemical staining of TMAs from the 115 patients included in the study was conducted. uPAR staining was found to be heterogeneously distributed within the tumour tissue, with most of the uPAR staining seen in the centre of the tumour islands, locating mainly to highly differentiated cells and somewhat less to the basaloid cells (Figure 1A). PAI-1 and uPA staining was equally distributed throughout the tumour tissue (Figure 1B and 1C). The staining was found mostly in the cytoplasm of the cancer cells, but some tumours also showed areas with membrane staining. All three antibodies also stained stromal cells to a varying degree.

**Figure 2:**
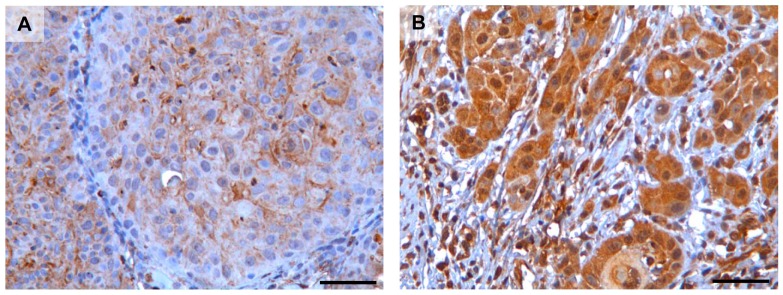
Cytoplasmic and membrane staining of uPAR. Representative photomicrographs of tissue microarray sections stained for uPAR, showing typical **A**) membrane and **B**) cytoplasmic localized staining of the tumour cells. Scalebar = 50 µm. Positive uPAR staining is seen as brown colour, nuclei are stained blue with haematoxylin.

**Figure 3:**
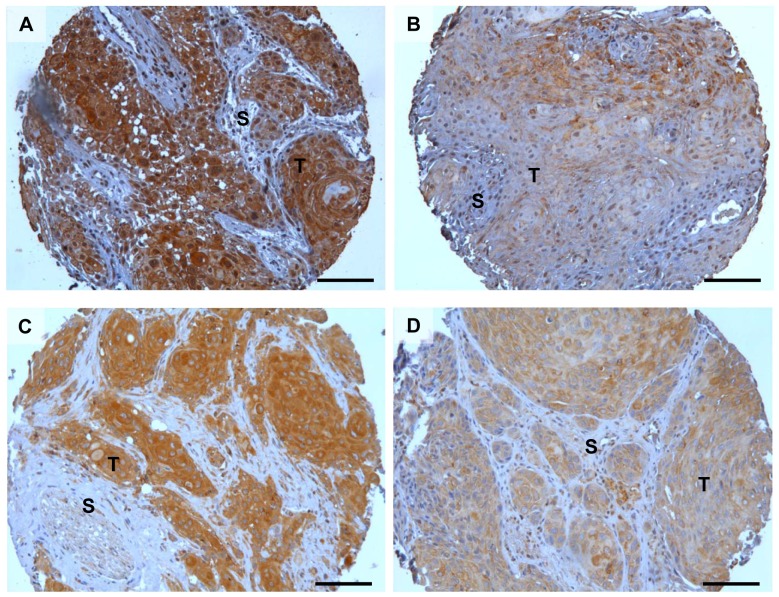
Staining intensity of uPAR and PAI-1 in OSCC. Representative photomicrographs of tissue microarray cores showing strong and weak staining for uPAR and PAI-1 in tumour islands: **A**) strong uPAR staining, **B**) weak uPAR staining, **C**) strong PAI-1 staining, and **D**) weak PAI-1 staining. Positive uPAR and PAI-1 staining is seen as brown colour, nuclei are stained blue with haematoxylin. Scalebar = 100 µm. T = Tumour. S = Stroma.

Figure 2 shows in more detail that uPAR staining could also be detected at the plasma membrane (Figure 2A) and in the nucleus (Figure 2B). The nuclear staining was not included in the scoring since it was only found in a few cores. Both uPAR and PAI-1 staining showed an inter-tumour variety that was easy to score, and typical examples of tumours with high and low scores are shown in figure 3. The average score for uPAR in the low expression group was 3.46 and 8.22 for the high expression group. For PAI-1, the average score for the low expression group was 3.74 and 6.80 for the high expression group.

**Figure 4:**
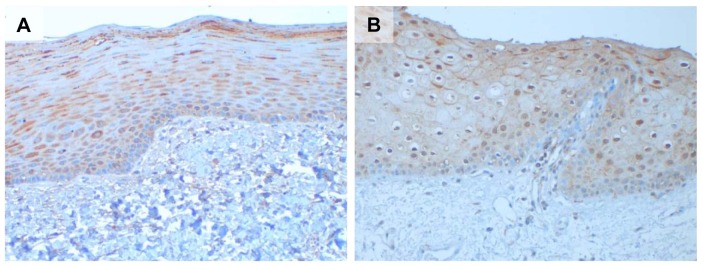
Staining of uPAR and PAI-1 in normal buccal mucosa tissue. Representative photomicrographs of normal buccal mucosa tissue showing weak staining for uPAR (**A**) and PAI-1 (**B**) in the epithelial layer. Positive staining is seen as brown colour, nuclei are stained blue with haematoxylin.

Staining of normal buccal mucosa tissue (n = 5) revealed that although there was some variation between the different samples, the staining intensity for both uPAR and PAI-1 was weak to moderate in the normal epithelium and the average score was 3.64 for uPAR and 4.42 for PAI-1 (Figure 4). The cut-off value used to distinguish between the low- and high expression groups was based on the median value of the final scores for each marker and was 5.63 for uPAR and 5.25 for PAI-1. Hence, the cut-off values for both uPAR and PAI-1 were higher than the average score of the normal tissue. Ki-67 staining was found exclusively in the nucleus (data not shown).

### Disease specific death in relation to biomarkers

**Figure 5:**
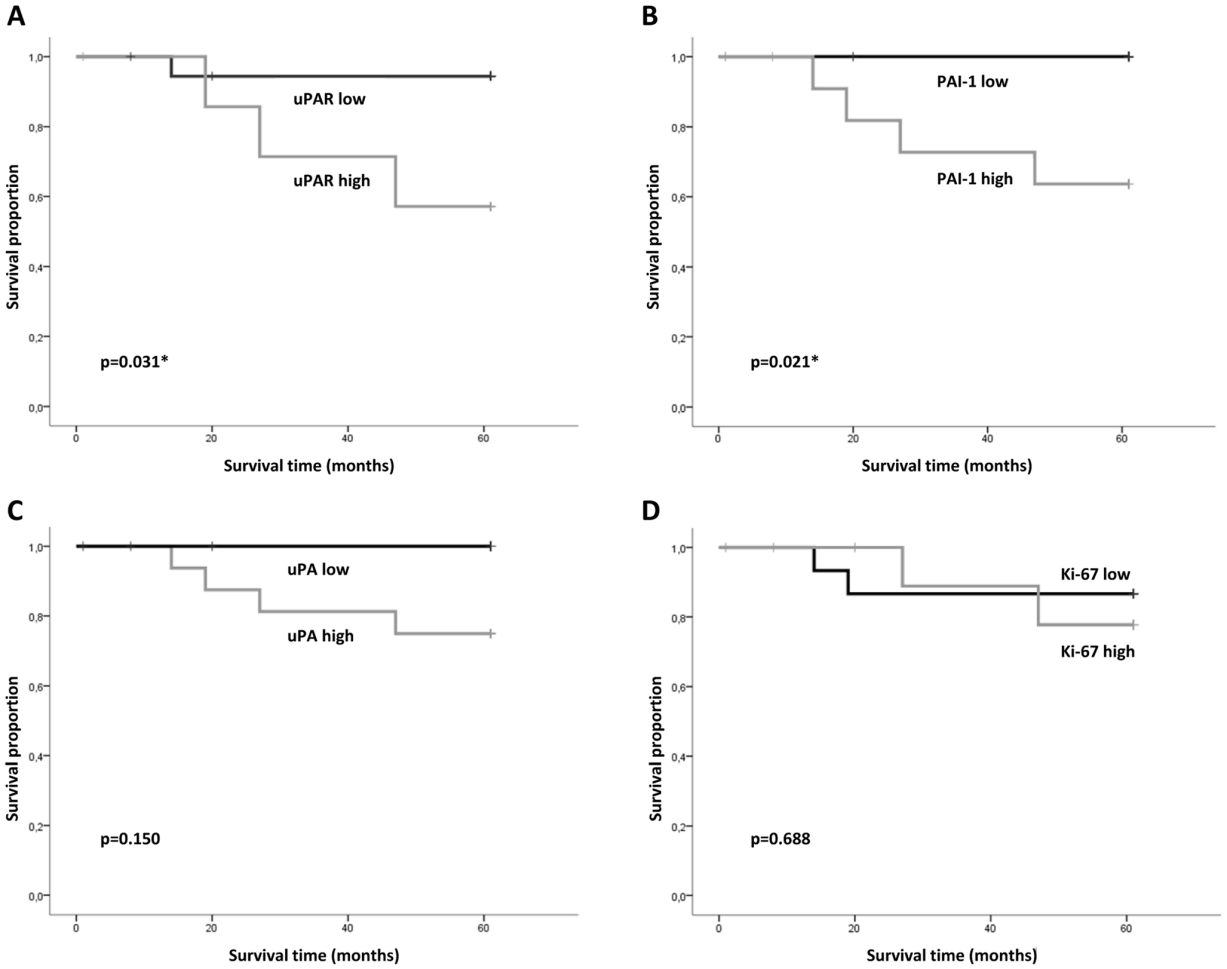
Disease specific survival of patients with T1N0 tumours. Kaplan-Meier survival plot showing probability for a disease specific survival based on **A**) uPAR, **B**) PAI-1, **C**) uPA and **D**) Ki-67 expression and related to months after diagnosis. Total number of patients included in the analysis was 27 for uPAR and Ki-67, and 26 for PAI-1 and uPA. *; p<0.05 was regarded as statistically significant.

The four biomarkers were tested in a univariate analysis for correlation with disease specific death within 5 years for all cases. None of the markers displayed any statistically significant association with disease specific death (data not shown). However, for patients with T1 tumours without lymph node metastasis (T1N0) at time of diagnosis, low uPAR expression was significantly (p = 0.031) associated with 5 year disease specific death (Figure 5A). A similar association was also found for expression of PAI-1 (p = 0.021)(Figure 5B), while neither uPA nor Ki-67 expression were associated with disease specific death (Figure 5C and D).

**Figure 6:**
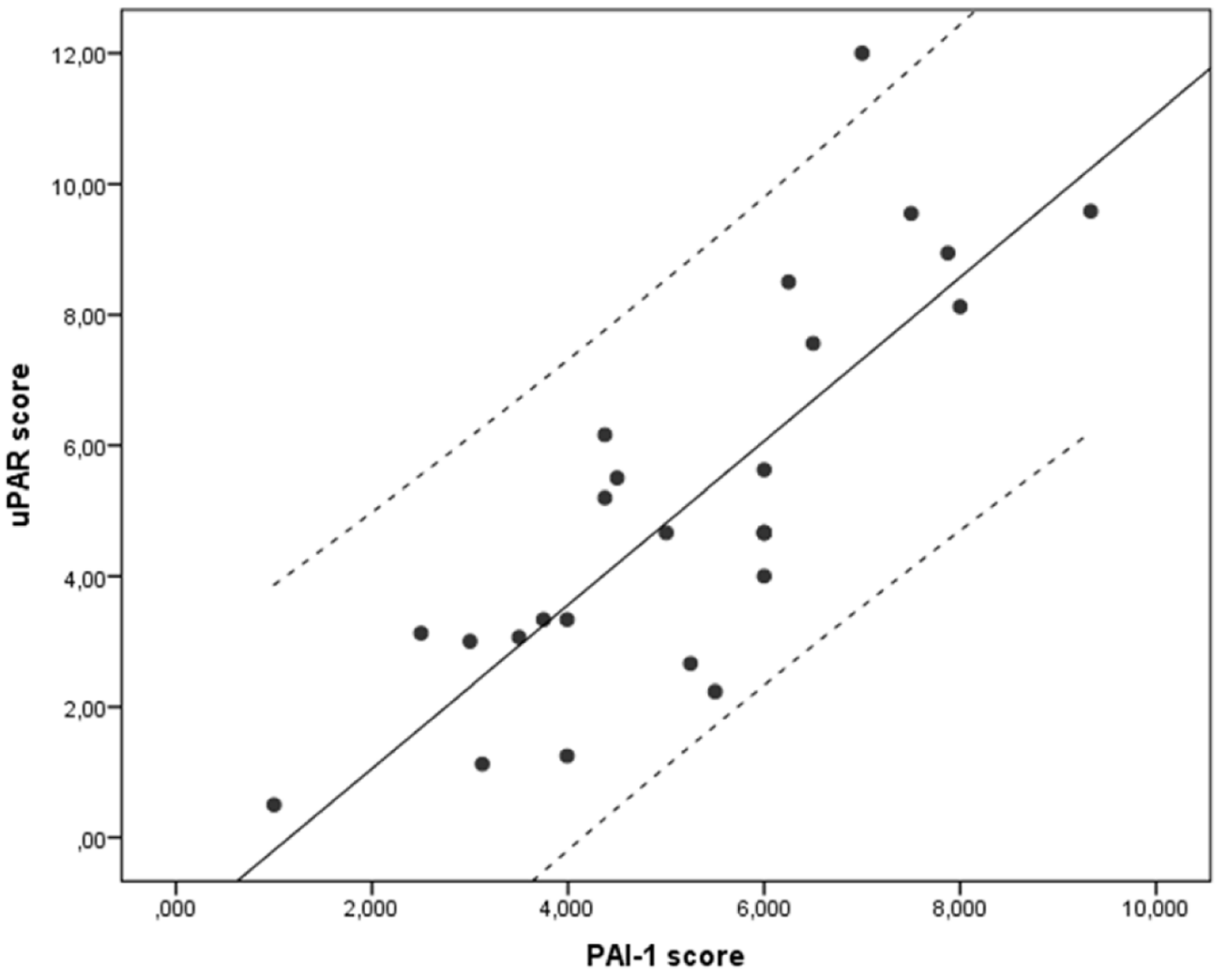
Correlation between uPAR and PAI-1 expression in T1N0 tumours. The correlation between the final scores of uPAR and PAI-1 in T1N0 tumours (N = 26) are presented in a Scatter plot (Spearman's Rho correlation coefficient = 0.566, p = 0.003). The regression line and the 95% confidence interval lines are indicated.

Furthermore, uPAR and PAI-1 expression correlated significantly with each other in the T1N0 tumours (Spearman's Rho correlation coefficient = 0.566, p = 0.003)(Figure 6). There was no statistically significant correlation between uPAR and PAI-1 when analysing the whole cohort (Spearman's Rho correlation coefficient = 0.145, p = 0.127). In order to assess for confounding factors, the distribution of low and high expression of uPAR and PAI-1 in relation to gender, tumour differentiation and the OSCC risk factors smoking and alcohol consumption, were analysed by a Pearson's Chi square test (Table 2). None of the factors were found to be correlated with the expression levels of uPAR or PAI-1.

**Table 2:** Distribution of low and high expression of uPAR and PAI-1 in relation to gender, tumour differentiation and the known OSCC risk factors smoking and alcohol consumption.

	uPAR			PAI-1		
	low	high	p*	low	high	p*
Gender						
Men	11 (58%)	3 (38%)	0.333	7 (54%)	6 (46%)	0.695
Women	8 (42%)	5 (62%)		6 (46%)	7 (54%)	
Tumour differentiation						
Well	10 (53%)	3 (38%)	0.556	6 (46%)	7 (54%)	0.584
Moderate	8 (42%)	5 (62%)		6 (46%)	6 (46%)	
Poor	1 (5%)	0 (0%)		1 (8%)	0 (0%)	
Smoking						
Never/previous	10 (53%)	2 (25%)	0.187	5 (38%)	6 (46%)	0.691
Smoker/unknown^1^	9 (47%)	6 (75%)		8 (62%)	7 (54%)	
Alcohol						
Never/<once a week	14 (74%)	5 (62%)	0.561	8 (62%)	10 (77%)	0.395
>once a week/daily/unknown^2^	5 (26%)	3 (38%)		5 (38%)	3 (23%)	

Total number of patients included in the analysis was 27 for uPAR and 26 for PAI-1.

*Pearson's Chi square test. p<0.05 was regarded as statistically significant.

1 Number of unknown is 1.

2 Number of unknowns is 3.

According to the pathology reports, the resection margins were clear for all of the T1N0 patients, except for four patients where the available information was inconclusive. All of these four patients survived more than 5 years. Eight of the T1N0 patients were treated with surgery only, the remaining received both surgery and radiotherapy. Two thirds of the T1N0 tumours were localized to the mobile tongue, while the remaining tumours were localized to the floor of mouth (N = 3), gingival rim (N = 3), bucca (N = 2), or soft palate (N = 1). All of the patients suffering a disease specific death (N = 4), died due to incurable lymph node metastasis. Two of those had elective neck dissection as part of the primary surgical treatment, while the other two did not. Hence, there was no correlation between surgical margins, tumour localization or treatment, with outcome or expression of uPAR or PAI-1.

## Discussion

Although the prognosis in OSCC is mainly determined by the stage of the tumour at presentation [2], [6], there is a need for reliable prognostic biomarkers that can be used for stratification of treatment options within subgroups of patients.

For OSCC in general, patients with small tumours have a better prognosis than patients with more advanced disease. Small tumours can however behave aggressively, and also in cases where no lymph node metastasis are found at diagnosis, the outcome is unpredictable [1]. The treatment options are surgery and/or radiotherapy. The treatment has major side effects that often reduce the patients' quality of life permanently. For patients with T1 tumours without lymph node metastasis (T1N0) the prognosis is particularly good. Nevertheless, the challenge is to cure the patient without overtreating. In Scandinavia, the treatment of choice for this group is surgery in the majority of cases. However, the fact that a large number of occult metastases are found in patients classified as N0 [7], [8], [38], prompts the need for tools to choose between “watchful waiting” or more extensive treatment. Advanced surgery with elective neck dissection is often performed or post-surgery radiotherapy is given because surgeons do not dare to refrain from treatment. The results provided in this study show that uPAR and PAI-1 correlate with disease specific death for patients with T1N0 tumours (Figure 5), and thus are good candidates for biomarkers that could aid in the decision-making.

In our samples, most of the uPAR, PAI-1 and uPA staining were found to be cytoplasmic or at the cell membrane (Figure 1). This is in accordance with the previously described localizations of these proteins [12], [18], [27], [29], [39]. In some patients, a few cells displayed nuclear uPAR localization (Figure 2). Similar nuclear immunoreactivity has been reported in pancreatic cancer [30], however the significance of this observation has not been determined. Thus, further studies are needed to clearly demonstrate nuclear localization of uPAR and the role it might have in the nucleus. uPAR expression was also significantly correlated with PAI-1 expression in T1N0 tumours (figure 6). This is in partial agreement with results from Lindberg et al. who analysed 20 cases of incipient OSCC and found that both uPAR and PAI-1, together with laminin γ2, were expressed in early invasive OSCC [18]. Using the R2 anti-uPAR antibody, they also report uPAR staining in stromal macrophages and fibroblasts surrounding tumours with low grade of invasion. In tumours with a higher grade of invasion, but not diffuse invasion, uPAR expression was found in both stromal- and cancer cells. In contrast, PAI-1 was found only in the cancer cells and not in any stromal cells. Thus, they suggest that PAI-1 is a better marker for initial OSCC invasion than uPAR. We stained normal buccal mucosa tissue and found that both uPAR and PAI-1 were weakly expressed in the epithelial layer. The average score of both were lower than the cut-off values used to separate the low and the high expressing tumours of both markers. Thus, the level of uPAR and PAI-1 in the tumours belonging to the low expression group is similar to the levels in normal buccal mucosa tissue. Nozaki et al. analysed 34 primary oral cancers and found that both uPAR (using the #3936 anti-uPAR antibody) and PAI-1 (using the MAI-11 anti-PAI-1 antibody) were expressed in 29.4% of the cases [12]. Although they found that fewer tumours were positive for uPAR and PAI-1, they also found a significant correlation between the expression of these proteins and mode of invasion. In our study, the use of TMA did not allow the evaluation of invasion pattern, hence further studies on whole tumour sections are needed in order to analyse this association in our material.

uPAR has previously been reported to correlate with overall 5 year survival in a Japanese cohort of 54 patients [13]. Bacchiocchi et al. also found low uPAR expression to correlate with increased overall survival in histological well differentiated OSCC tumours (G1, 2003 WHO classification), but not in more poorly differentiated (G2 or G3) tumours [39]. Their cohort consisted of 189 patients where 77 were G1 tumours. Of these, 41 were classified as TNM stage I, thus more than half of the cases were T1N0M0. Thus, together with our results, this strongly suggests that uPAR should be analysed further as a prognostic biomarker for early stage tumours in OSCC.

PAI-1 has been proposed as a prognostic marker linked to poor prognosis in several cancers [40], including in OSCC [16], [18]. In breast cancer, PAI-1 together with uPA, have been convincingly shown to be strong prognostic markers, have recently reached “level-of-evidence 1” and are recommended in clinical use as a stratification parameter for treatment of node-negative breast cancer [41]. In their study, the determination of uPA and PAI-1 levels was done by certified ELISA tests on extracts of fresh-frozen primary tumour tissue and not by IHC [42].

The Ki-67 proliferation marker is in use to predict prognosis in several cancer types, although with some controversy [5], [43]. Also for OSCC there is no consensus for Ki-67 being a prognostic marker. Gonzalez-Moles et al. showed in a Spanish cohort of 65 patients that Ki-67 lacks prognostic value [44], whereas it was recently reported that high Ki-67 was a marker for good prognosis in a Canadian cohort of 121 patients [45]. Our data showed no statistically significant correlation with Ki-67 and survival in any of the subgroups analysed. As stated in a review by Schliephake [10], only 12 out of 23 reports on proliferation markers were associated with prognosis. Therefore, high Ki-67 score should not be used to support a decision for further treatment of OSCC patients.

In conclusion, our results show that patients with T1N0 tumours with low expression of uPAR and PAI-1 have decreased risk of disease specific death. However, since our present cohort of these tumours is relatively small, further studies on larger cohorts must be performed in order to determine the use of uPAR and PAI-1 as prognostic markers and tools for decision-making with regards to treatment options.

## Supporting Information

Figure S1 **Specificity of the anti-uPAR antibody (#3936).** Photomicrographs of tissue microarray sections stained for uPAR. **A**) The uPAR antibody was incubated in the presence of recombinant His-tagged uPAR, antibody-antigen complexes were removed by precipitation and remaining unbound material was used for immunohistochemical staining of the tissue microarray section. **B**) The uPAR antibody received the same pre-treatment as in a), except that the antibody was incubated without His-tagged recombinant uPAR. **C**) The antibody received no pre-treatment. **D**) Western blot showing uPAR expression in whole cell lysates from the cell lines U937 (human) and GD25 (murine). Lane 1: non-stimulated U937 cells, lane 2: U937 cells stimulated with 200 nM PMA for 24 hours, lane 3: U937 cells stimulated with 200 nM PMA for 48 hours, lane 4: GD25 cells, lane 5: GD25 cells stably overexpressing human uPAR. Left panel: The uPAR antibody received the same pre-treatment as described in b). Right panel: The uPAR antibody was pre-incubated with the presence of recombinant His-tagged uPAR, as described in a).
(TIF)

Figure S2 **Specificity of the anti-uPA antibody (Ab24121).** Photomicrographs of pancreatic cancer sections immunohistochemically stained for uPA. **A**) Strong uPA expression in pancreatic cancer with lack of staining in nerve (asterix). **B**) uPA expression in pancreatic cancer, but not in normal ducts (asterix). **C**) uPA expression in pancreatic cancer, but negative in benign pancreatic tissue (asterix).
(TIF)

Figure S3 **Specificity of the anti-PAI-1 antibody (BT-BS3505).** Placenta tissue was stained with two different PAI-1 antibodies; #3785, used to stain the TMAs, and BS3505. **A**: Cytotrophoblasts present in the maternal plate of the placenta were positively stained using the anti-human PAI-1 antibody (#3785), while the surrounding tissue was negative. **B**: Placental tissue stained with the anti-human PAI-1 antibody (BT-BS3505) showed similar positive staining patterns of the cytotrophoblasts in the placenta plate as the #3785 antibody.
(TIF)

Table S1 **Disease specific death (DSD) for all cases (N = 115) in relation to clinicopathological variables.**
(PDF)

Information S1 **Specificity of antibodies.** Materials, methods and results for the verification of the specificities of the antibodies used.
(PDF)
