# Supplementary figures and images for: Urokinase Plasminogen Activator Receptor (uPAR) and Plasminogen Activator Inhibitor-1 (PAI-1) Are Potential Predictive Biomarkers in Early Stage Oral Squamous Cell Carcinomas (OSCC)
Source: PLoS One. 2014 Jul 7;9(7):e101895. doi: 10.1371/journal.pone.0101895 (PMC4084992; doi:10.1371/journal.pone.0101895)

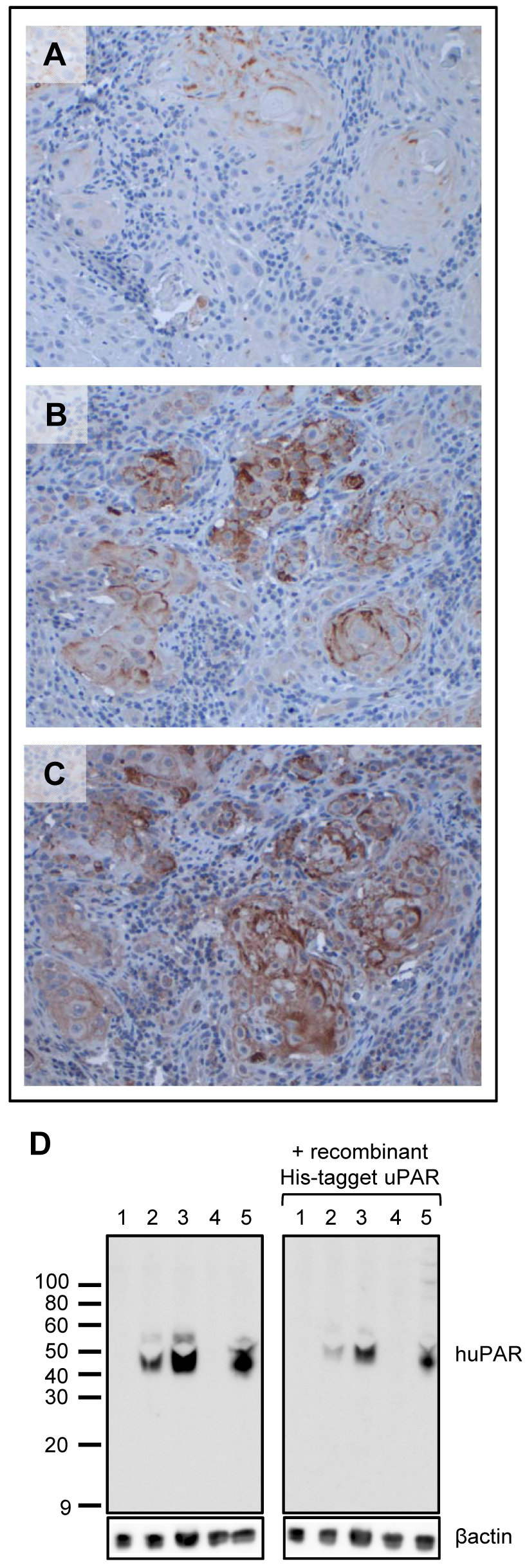

Supplement: Figure S1 — Specificity of the anti-uPAR antibody (#3936). Photomicrographs of tissue microarray sections stained for uPAR. A) The uPAR antibody was incubated in the presence of recombinant His-tagged uPAR, antibody-antigen complexes were removed by precipitation and remaining unbound material was used for immunohistochemical staining of the tissue microarray section. B) The uPAR antibody received the same pre-treatment as in a), except that the antibody was incubated without His-tagged recombinant uPAR. C) The antibody received no pre-treatment. D) Western blot showing uPAR expression in whole cell lysates from the cell lines U937 (human) and GD25 (murine). Lane 1: non-stimulated U937 cells, lane 2: U937 cells stimulated with 200 nM PMA for 24 hours, lane 3: U937 cells stimulated with 200 nM PMA for 48 hours, lane 4: GD25 cells, lane 5: GD25 cells stably overexpressing human uPAR. Left panel: The uPAR antibody received the same pre-treatment as described in b). Right panel: The uPAR antibody was pre-incubated with the presence of recombinant His-tagged uPAR, as described in a). (TIF) [file pone.0101895.s001.tif]

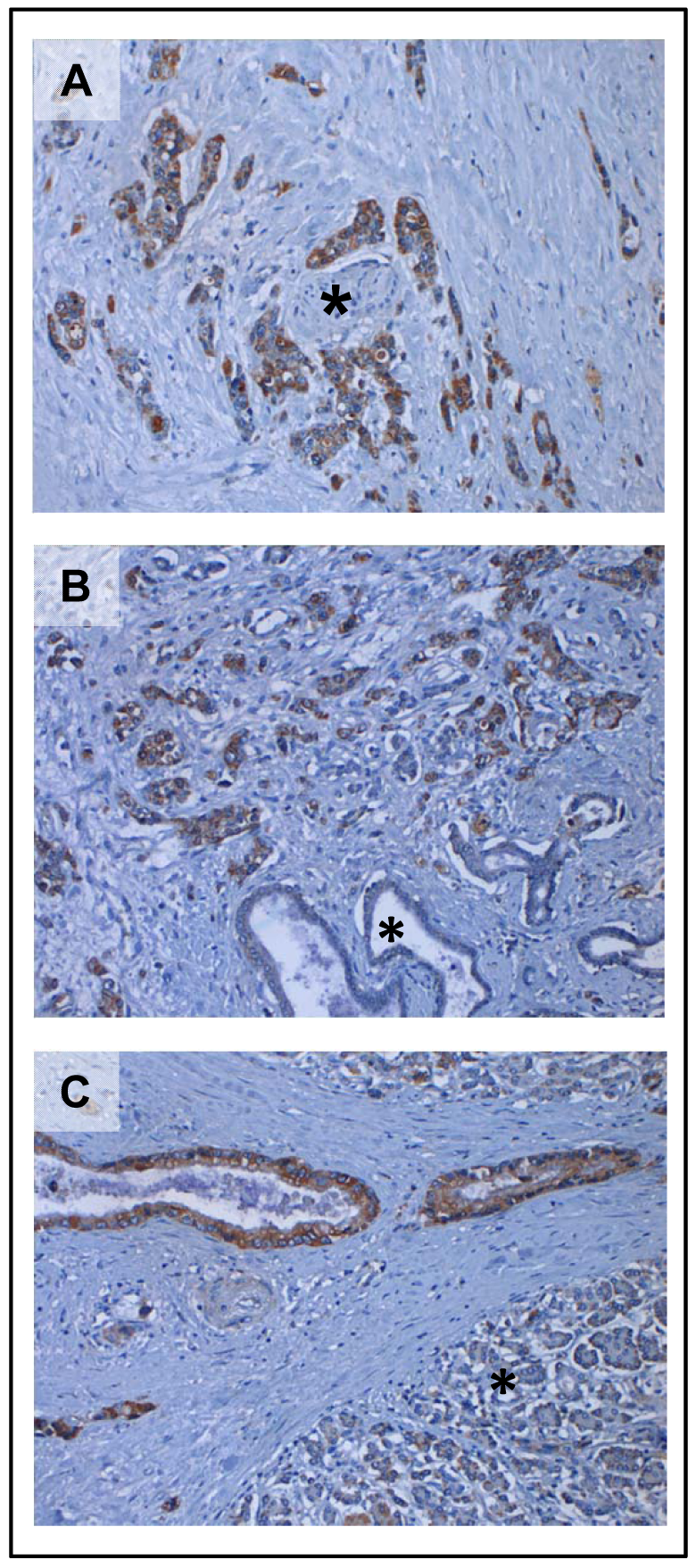

Supplement: Figure S2 — Specificity of the anti-uPA antibody (Ab24121). Photomicrographs of pancreatic cancer sections immunohistochemically stained for uPA. A) Strong uPA expression in pancreatic cancer with lack of staining in nerve (asterix). B) uPA expression in pancreatic cancer, but not in normal ducts (asterix). C) uPA expression in pancreatic cancer, but negative in benign pancreatic tissue (asterix). (TIF) [file pone.0101895.s002.tif]

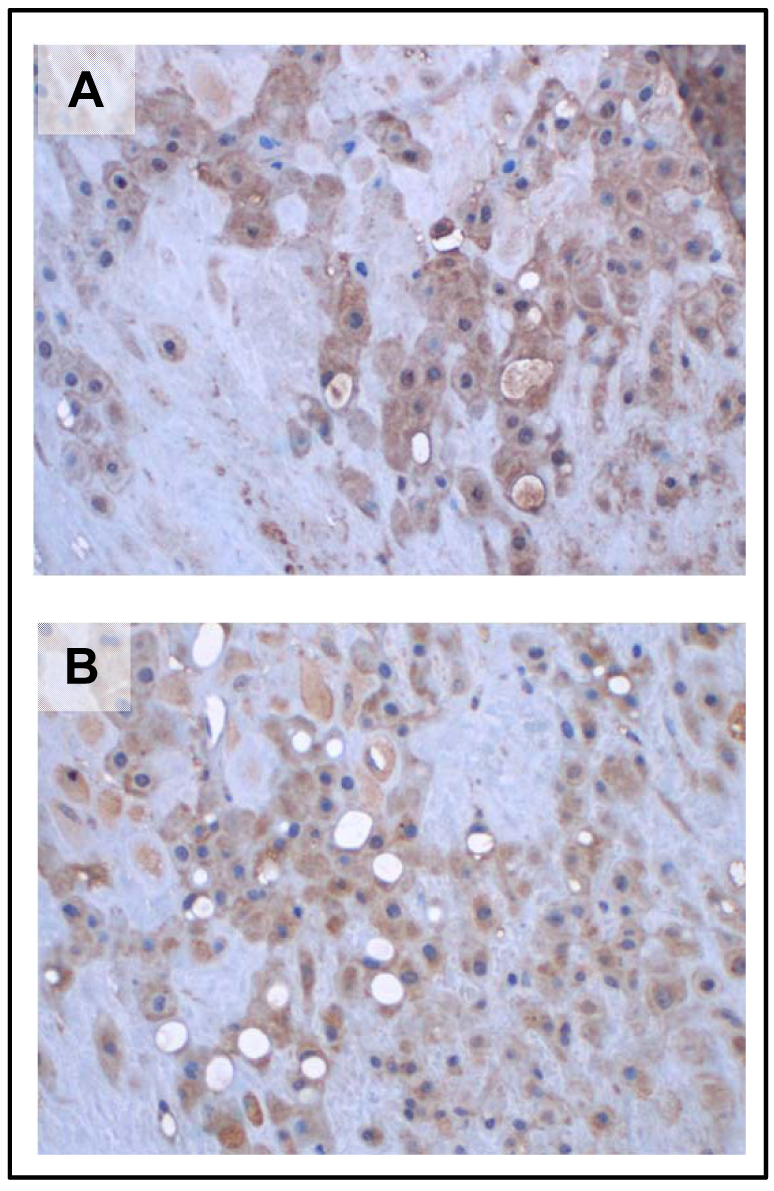

Supplement: Figure S3 — Specificity of the anti-PAI-1 antibody (BT-BS3505). Placenta tissue was stained with two different PAI-1 antibodies; #3785, used to stain the TMAs, and BS3505. A: Cytotrophoblasts present in the maternal plate of the placenta were positively stained using the anti-human PAI-1 antibody (#3785), while the surrounding tissue was negative. B: Placental tissue stained with the anti-human PAI-1 antibody (BT-BS3505) showed similar positive staining patterns of the cytotrophoblasts in the placenta plate as the #3785 antibody. (TIF) [file pone.0101895.s003.tif]
