# Supplementary material for: Urokinase Plasminogen Activator Receptor (uPAR) and Plasminogen Activator Inhibitor-1 (PAI-1) Are Potential Predictive Biomarkers in Early Stage Oral Squamous Cell Carcinomas (OSCC)
Source: PLoS One. 2014 Jul 7;9(7):e101895. doi: 10.1371/journal.pone.0101895 (PMC4084992; doi:10.1371/journal.pone.0101895)
Supplement: Table S1 — Disease specific death (DSD) for all cases (N = 115) in relation to clinicopathological variables. (PDF) [file pone.0101895.s004.pdf]

Table S1. Disease specific death (DSD) for all cases (N=115) in relation to clinicopathological variables.

	Patients N (% of total)	5-year DSD N (% of total)	DSD p
**Gender**			
Male	64 (56%)	27 (42%)	0.557
Female	51 (44%)	19 (37%)	
**Tumour differentiation**			
Well	46 (40%)	15 (33%)	0.187
Moderate	58 (50%)	25 (43%)	
Poor	11 (10%)	6 (55%)	
**Tumour size**			
T1	39 (34%)	7 (18%)	**<0.001***
T2	42 (37%)	12 (29%)	
T3	8 (7%)	6 (75%)	
T4	22 (19%)	17 (77%)	
Unknown	4 (3%)	4 (100%)	
**Lymph node status**			
N0	72 (63%)	20 (27%)	**<0.001***
N+	31 (27%)	20 (65%)	
Unknown	12 (10%)	6 (50%)	

*; p<0.05 was regarded as statistically significant
