# Supplementary material for: Urokinase Plasminogen Activator Receptor (uPAR) and Plasminogen Activator Inhibitor-1 (PAI-1) Are Potential Predictive Biomarkers in Early Stage Oral Squamous Cell Carcinomas (OSCC)
Source: PLoS One. 2014 Jul 7;9(7):e101895. doi: 10.1371/journal.pone.0101895 (PMC4084992; doi:10.1371/journal.pone.0101895)
Supplement: Information S1 — Specificity of antibodies. Materials, methods and results for the verification of the specificities of the antibodies used. (PDF) [file pone.0101895.s005.pdf]

Supporting information S1

Materials and methods

Preadsorption of antibodies

The anti-uPAR antibody was tested by preadsorption of the antibody with His-tagged recombinant human uPAR. In brief, 10 μg of mouse monoclonal anti-human uPAR antibody (#3936, Sekisui Diagnostica, Stamford, CT, USA) was mixed with a 2x molar surplus (4 μg) of recombinant His-tagged human uPAR protein (#807-UK-100, R&D Systems, Minneapolis, MN) in assay buffer (1 x PBS, 1% BSA, 0.3% Tween, pH 7,4) and incubated overnight at 4°C to allow binding. Talon Superflow His-tag purification resin (GE Healthcare Bio-Sciences AB, Uppsala, Sweden) in binding buffer (50mM sodium phosphate, 300 mM NaCl, pH 7.4) was then added to precipitate complexes of antibodies and His-tagged protein. The supernatant containing the unbound antibodies was recovered by centrifugation and used for IHC and Western blotting. For the negative control, the antibody was treated identically, except that the recombinant uPAR protein was omitted.

Cells

GD25 cells [1-3] stably overexpressing human uPAR were cultured in DMEM (D6046, Sigma Aldrich, St. Louis, USA) supplemented with 10% FBS (F7524, Sigma Aldrich, St. Louis, USA) (Svineng, unpublished results). The human leukemic monocyte cell line U937 was a kind gift from K. Nilsson (Department of Pathology, University of Uppsala, Sweden) and verified by STR analysis performed at the Centre of Forensic Genetics at the University of Tromsø, Norway. The U937 cells were cultured in $NaHCO_3$-buffered RPMI-1640 (R8758, Sigma Aldrich, St. Louis, USA) supplemented with 10% FBS (F7524, Sigma Aldrich, St. Louis, USA). Both cell lines were cultured at 37^oC, 5% CO_2 in a humidified incubator.

Western blotting

GD25 cells were detached using trypsin (0.25% in PBS with 0.05% Na_2EDTA), counted, and 3 x 10^5 cells were seeded per well in a 6-well plate and incubated for 24 hours. U937 cells were counted and $3x10^5$ cells were added per well to a 6-well plate. U937 cells were either non-stimulated or added PMA (P8139, Sigma Aldrich, St. Louis, USA). PMA stimulation was

performed by adding PMA to RPMI-1640 containing 10% heat inactivated serum to a final concentration of 200 nM. Cells were then incubated for either 24 hours or 48 hours. Non-stimulated cells were incubated in RPMI-1640 with 10% heat inactivated serum. Cells were harvested by scraping in 200 µl sample buffer (0.05 M Tris-HCl pH 6.8, 2% SDS, 10% glycerol, 0.1% bromphenol blue), sonicated and boiled before the samples were loaded onto NuPAGE Novex 4%-12% Bis-Tris gels (Invitrogen, Eugene, USA), and subjected to non-reducing SDS-PAGE. Proteins were blotted onto PVDF membranes (Millipore Corp., Bedford, USA), and blocking was done with 5% non-fat dry milk in Tris-buffered saline (150 mM NaCl, 20 mM Tris, pH 7.4) supplemented with 0.1% Tween 20. Membranes were incubated with primary antibody recognizing human uPAR (#3936), 1:500 dilution 4°C ON. The antibody received pre-treatment as described above (see "preadsorption of antibodies"). The primary antibody was detected using a 1:50.000 dilution of the HRP conjugated anti-mouse IgG (#A2554, Sigma Aldrich, St. Louis, USA), 1 hour RT. Equal loading was controlled by re-probing for β-actin (A3854), 1:100.000 dilution, 1 hour RT. Western blotting Luminol Reagent (Santa Cruz Biotechnology Inc., USA) was used for antibody detection, and images were obtained using the Fujifilm LAS-4000 imaging system (Fujifilm, Tokyo, Japan).

IHC staining for uPA

Pancreas cancer sections were treated as previously described (see "immunohistochemistry") in the materials and methods section. The anti-uPA antibody (Ab24121, Abcam Inc., Cambridge, MA, USA) was diluted 1:20 in PBS ^{w}/1.5% goat serum and incubated at room temperature for 1 hour. Detection was performed as described in table 1.

IHC staining for PAI-1

Sections of human placenta were stained with two different antibodies for PAI-1 to verify similar staining patterns. Sections were treated as previously described (see "immunohistochemistry") in the materials and methods section. For the anti-PAI-1 antibody (#3785, Sekisui Diagnostica Stamford, CT, USA) the procedure was followed according to the manufacturer's protocol. Changes made to the protocol are listed in table 1 (buffers used, and the antibody dilution). The HIER procedure and buffers for the BT-BS3505 (Nordic BioSite, Täby, Sweden) antibody are described in table 1. For placenta tissue the antibody was diluted 1:75 in PBS ^{w}/1.5% goat serum and incubated at 4°C overnight.

Results

Verification of the anti-uPAR antibody

As suggested by Bordeaux et al.[4], antibodies could be tested for specificity and selectivity by various methods. To verify the mouse monoclonal anti-human uPAR antibody (#3936, Sekisui Diagnostica, Stamford, CT, USA) we first performed IHC using the #3936 antibody preadsorbed with recombinant His-tagged human uPAR (R&D Systems, Minneapolis, MN). A marked decrease in staining was observed using the preadsosorbed antibody compared to the control where no His-tagged uPAR was added to the preadsorption mix (Figure S1).

The specificity of the antibody was also tested by Western blotting of whole cell lysates. Bands of the expected size corresponding to glycosylated uPAR were detected in both PMA stimulated U937 cells and GD25 cells overexpressing human uPAR, and no additional bands could be detected (Figure S1). The strength of the signal was markedly reduced when the antibody had been preadsorbed with recombinant uPAR.

Verification of the anti-uPA antibody

Pancreatic cancer tissue has been shown to stain positively for uPA [5]. The staining pattern obtained using the ab24121 anti-uPA antibody was similar to that reported by Nielsen et al. with specific staining of tumors cells and negative staining in benign pancreatic- and nerve tissue (Figure S2) [5]. In addition, the ab24121 antibody has previously been shown to stain uPA in esophageal adenocarcinoma [6]. A different anti-uPA antibody (Ab64520, Abcam Inc., Cambridge, MA, USA) was also tested on the same tissue to verify similar staining patterns, but the antibody displayed less specificity than the ab24121 antibody and was therefore not included in the final results (results not shown).

Verification of the anti-PAI-1 antibody

As shown by others, placenta cytotrophoblasts express PAI-1 [7,8]. In accordance with this, we found that the BT-BS3505 anti-PAI-1 antibody stained the cytotrophoblasts in the placenta plate, while the surrounding stromal tissue was negative. Similar staining patterns and intensities were also found using the monoclonal anti-PAI-1 antibody (#3785, Sekisui Diagnostica) (Figure S3). The #3785 antibody has previously been shown to stain PAI-1 in breast cancer tissue [9,10].

References

1. Fassler R, Pfaff M, Murphy J, Noegel AA, Johansson S, et al. (1995) Lack of beta 1 integrin gene in embryonic stem cells affects morphology, adhesion, and migration but not integration into the inner cell mass of blastocysts. J Cell Biol 128: 979-988.
2. Wennerberg K, Lohikangas L, Gullberg D, Pfaff M, Johansson S, et al. (1996) Beta 1 integrin-dependent and -independent polymerization of fibronectin. J Cell Biol 132: 227-238.
3. Svineng G, Johansson S (1999) Integrin subunits (beta)1C-1 and (beta)1C-2 expressed in GD25T cells are retained and degraded intracellularly rather than localised to the cell surface. J Cell Sci 112 (Pt 24): 4751-4761.
4. Bordeaux J, Welsh A, Agarwal S, Killiam E, Baquero M, et al. (2010) Antibody validation. Biotechniques 48: 197-209.
5. Nielsen A, Scarlett CJ, Samra JS, Gill A, Li Y, et al. (2005) Significant overexpression of urokinase-type plasminogen activator in pancreatic adenocarcinoma using real-time quantitative reverse transcription polymerase chain reaction. J Gastroenterol Hepatol 20: 256-263.
6. Goscinski MA, Suo Z, Florenes VA, Vlatkovic L, Nesland JM, et al. (2008) FAP-alpha and uPA show different expression patterns in premalignant and malignant esophageal lesions. Ultrastruct Pathol 32: 89-96.
7. Feinberg RF, Kao LC, Haimowitz JE, Queenan JT, Jr., Wun TC, et al. (1989) Plasminogen activator inhibitor types 1 and 2 in human trophoblasts. PAI-1 is an immunocytochemical marker of invading trophoblasts. Lab Invest 61: 20-26.
8. Hu ZY, Liu YX, Liu K, Byrne S, Ny T, et al. (1999) Expression of tissue type and urokinase type plasminogen activators as well as plasminogen activator inhibitor type-1 and type-2 in human and rhesus monkey placenta. J Anat 194 (Pt 2): 183-195.
9. Hildenbrand R, Arens N (2004) Protein and mRNA expression of uPAR and PAI-1 in myoepithelial cells of early breast cancer lesions and normal breast tissue. Br J Cancer 91: 564-571.
10. Elzi DJ, Lai Y, Song M, Hakala K, Weintraub ST, et al. (2012) Plasminogen activator inhibitor 1--insulin-like growth factor binding protein 3 cascade regulates stress-induced senescence. Proc Natl Acad Sci U S A 109: 12052-12057.
